# Medication-related calls received by a national telenursing triage and advice service in Australia: a retrospective cohort study

**DOI:** 10.1186/s12913-017-2135-1

**Published:** 2017-03-14

**Authors:** Ling Li, Rebecca Lake, Magdalena Z. Raban, Mary Byrne, Maureen Robinson, Johanna Westbrook, Melissa T. Baysari

**Affiliations:** 1 Centre for Health Systems and Safety Research, Australian Institute of Health Innovation, Faculty of Medicine and Health Sciences, Level 6, 75 Talavera Road, Macquarie University, Sydney, NSW 2109 Australia; 2 Healthdirect Australia, Sydney, NSW Australia; 3 Department of Clinical Pharmacology & Toxicology, St Vincent’s Hospital, Sydney, Australia

**Keywords:** Nursing, Telenursing, Telephone helpline, Nurse triage, Medication queries, Helpline, Telephone advice

## Abstract

**Background:**

Telenursing triage and advice services are increasingly being used to deliver health advice. Medication-related queries are common, however little research has explored the medication-related calls made to these services. The aim of this study was to examine the profile of medication-related calls to a national telenursing triage and advice service and the medications involved.

**Methods:**

This was a retrospective cohort study of medication-related calls received by Australia’s national helpline (healthdirect helpline) in 2014, which provides free advice from registered nurses. We examined the volume of medication-related calls over time, user profiles for patients and callers, and call characteristics and we also investigated medications involved in the calls by their generic names and therapeutic classes.

**Results:**

Of 675,774 calls, 3.8% (*n* = 25,744) were medication-related, which was the largest category of calls. The average call length was 10 min. Over half of callers (55.4%) were advised to deliver self-care. Of 7,459 calls where the callers reported they did not know what to do prior to calling, 56.8% were advised to self-care and 3.5% were transferred to the Poisons Information Centre immediately. Of 1,277 calls where callers reported that they had originally intended to call an ambulance or attend an emergency department (ED), none were advised to do so. Advice most frequently requested was about analgesics and antipyretics, followed by non-steroidal anti-inflammatory agents.

**Conclusion:**

The telenursing triage and advice helpline offered quick and easily accessible advice, and provided reassurance to patients and callers with medication-related queries. The service also potentially diverted some patients from attending an ED unnecessarily.

## Background

Increasing demands on healthcare systems have led to changes in healthcare delivery [1]. Information and communication technologies (ICT) present an opportunity to re-organise the composition of teams who deliver care, and the processes of care delivery. Telehealth is one ICT that can change the way healthcare professionals interact with consumers and with one another [2].

Telenursing triage and advice services are increasingly being used to deliver health advice, and have been shown to have high levels of acceptability to callers [3]. The general premise is that patients can make a free, or local, call and receive advice from nurses for common problems. A trained advisor (supported by experienced nurses and paramedics) or a nurse, depending on the service, will usually collect caller details, and information about symptoms. Depending on the severity of the symptoms, the advisor or nurse may recommend self-care, transfer the call to another healthcare practitioner (e.g., general practitioner (GP)), or advise that the patient attends a health service in person (e.g., GP surgery, accident and emergency/emergency department (ED)). National, publicly available telephone based health services, such as nurse triage and advice services (e.g., National Health Service (NHS) 111 in England (previously NHS Direct), NHS 24 in Scotland, and healthdirect helpline in Australia), promise a range of benefits, including ease of access, fast responses, user anonymity and privacy.

For decades, nurses have performed the task of triaging patients to assess their level of care needs. More recently, there has been an increase in telephone-based triage by nurses, both in general medical practices and emergency departments. This practice has been effective in reducing doctor workload [4] and improving healthcare access [5, 6]. The safety and effectiveness of telephone triage by nurses has been demonstrated in Britain [7, 8] and New Zealand [9].

Medication prescription, therapy and advice form a large part of healthcare, and calls to these telephone lines often comprise medication queries. Callers regularly require quick advice about medications, such as actions to take when doses are omitted or taken accidentally, side effects, whether different medications will interact, or whether medications are safe for patients with certain conditions. Past research has focused on medication advice over the telephone targeting the public or healthcare professionals, provided by clinical pharmacologists, clinical pharmacists, poison specialists and clinical anaesthetists [10–16]. Literature around nurses’ provision of telephone based medication advice is, however, lacking.

Previous studies have also examined telephone based medication therapy management, adherence and prescriptions. An American study [17, 18] examined medication therapy management over the telephone, with positive results. In this case, clients with chronic illnesses who were taking two or more medications were provided with a telephone based medication review and received an action plan. The research team concluded that this telephone-based intervention was effective in solving medication-related health problems.

Research has shown that nurses are capable of safely and effectively handling general medical queries and providing advice [8, 19]. NHS Direct in the UK was staffed predominantly by nurses, and was highly utilised and viewed positively by the public [20].

Research on telephone consultation and triage helplines is expanding, with several studies and reviews published in the past decade [1, 4, 5, 7–9, 20, 21]. However, research examining the medication advice requested and given by such services is very limited. We identified no previous studies on telenursing advice related to medications. With the expanding use of telephone triage and advice services around the globe, the issue of telephone medication advice deserves further exploration.

## Methods

### Aims

This study had two main aims: 1) to examine the profile of medication-related calls, including characteristics of patients, callers and calls, and 2) to investigate the medications involved in these calls.

### Study design and setting

This retrospective cohort study examined medication-related calls received by the healthdirect helpline in Australia in 2014. Staffed by registered nurses, the healthdirect helpline provides free 24 h/seven day telephone health advice to people calling from four states and two territories, serving 54.9% of Australia’s total population [22]. The registered nurses do not provide diagnoses – only advice and information. The healthdirect helpline is funded by most of the states and the federal governments of Australia.

De-identified call records are collected routinely by the healthdirect helpline. The data includes demographics of callers and patients, the relationship between callers and patients, time of calls, callers’ original intentions before contacting the service (including not knowing what to do), care advice (the advice provided by nurses, also known as patients’ final dispositions in the healthdirect database) and the free text entered by nurses about a patient’s presenting problem. Nurses might transfer calls to NPS Medicine Wise for further medication information after providing care advice (this data was not available for this study).

Registered nurses document the presenting problem as described by a caller in a free text field. From the free text field, we identified medications involved and then coded medication names into their generic names and therapeutic classes based on the Monthly Index of Medical Specialties (MIMS) Australia database [23]. The MIMS database was used as it is a highly trusted and independent source of drug information used by healthcare professionals across Australia, New Zealand and Asia. MIMS classifies medications into 143 therapeutic classes, which fall into 22 broader classes (see Appendix). We focused on medication-related calls made in November 2014, given the large amount of manual checking involved in identifying medications from the free-text field.

### Data analysis

We conducted a detailed descriptive analysis of 2014 data over time, specifically examining the volume of medication-related calls, the profiles of patients and callers (including age, gender, cultural background and frequent users), and call characteristics (including time and date of calls, patient outcomes, and duration of calls). Based on the data extracted from the patient presenting problems, we analysed the frequency of medications by their generic names and therapeutic classes for the month of November.

## Results

### Medication-related calls over time

In 2014, there were 675,774 calls recorded in the healthdirect helpline database. The largest number of calls were made in January, followed by August, March and December. There were 416 call categories. Among these calls, 25,744 calls (3.8%) for 23,254 patients were medication-related, which represented the largest single category of calls in the database. Other frequent call categories included abdominal pain/discomfort (3.0%), paediatric vomiting (3.0%), chest pain/discomfort (2.8%), paediatric trauma - head (1.9%), paediatric fever (1.7%) and paediatric colds (1.5%).

**Fig. 1:**
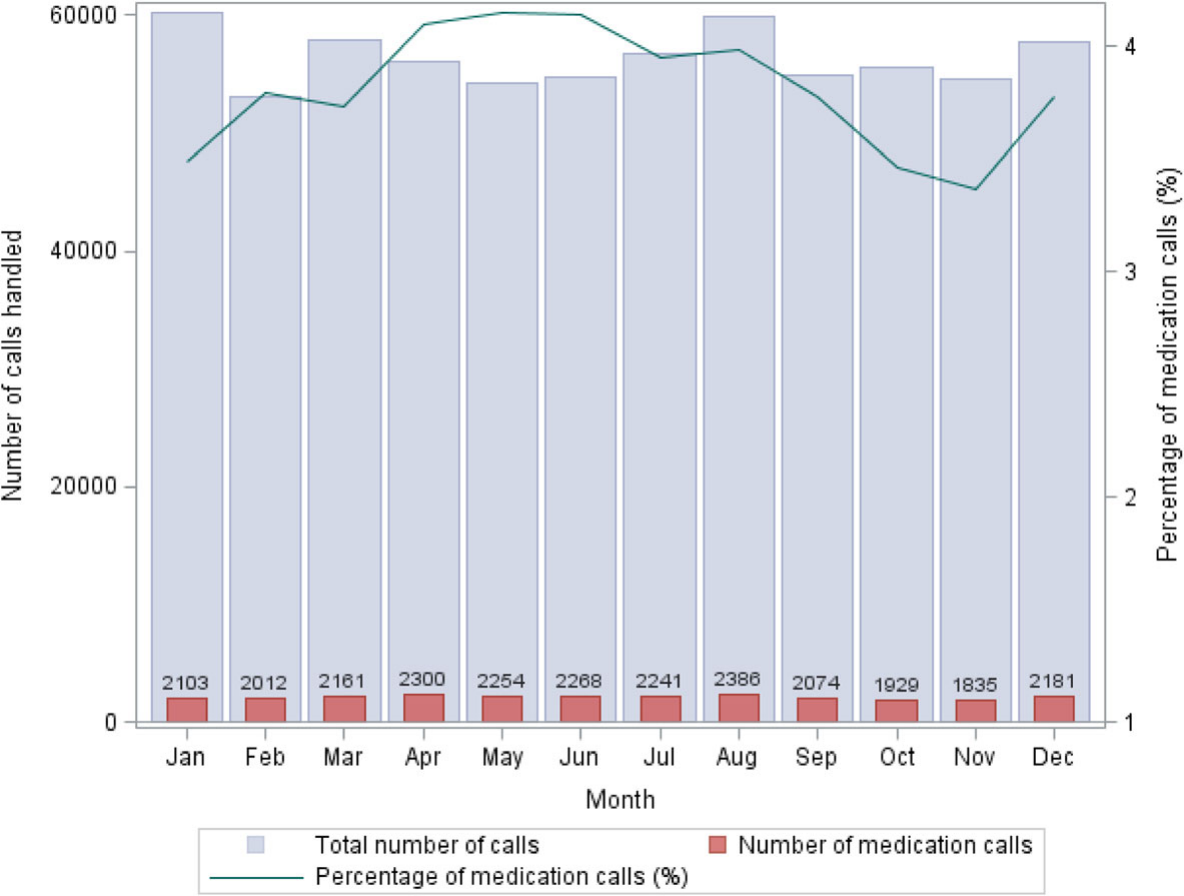
Healthdirect helpline calls (*n* = 675,774) and medication-related calls (*n* = 25,744) over time in 2014

**Fig. 2:**
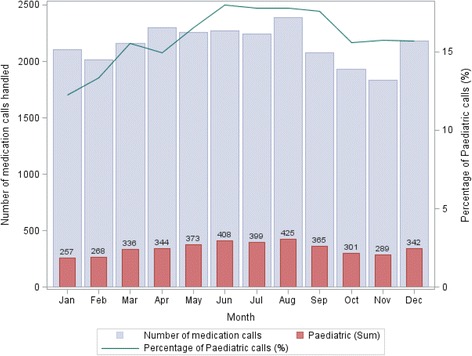
Paediatric and adult medication-related calls (*n* = 4,078) over time

The percentage of medication-related calls received in each month varied between 3.4 and 4.2%. April, May and June had the highest proportion of medication-related calls (Fig. 1). Of all medication-related calls, 15.8% related to paediatric medications (*n* = 4078, Fig. 2). The colder months (from June to September) had the highest percentage of paediatric medication calls.

### Patient characteristics

The majority of patients (75.1%, *n* = 17,467) were female. Patients aged between 26 and 45 years were the most frequent clients of the service (35.5%, *n* = 9,134 calls), followed by those aged 46–65 years (21.0%, *n* = 5,397 calls), as shown in Table 1.

**Table 1:** Medication-related calls by patient age

Patient age	Number of calls	Percentage (%)
≤14 years	4079	15.8
15-25 years	3085	12.0
26-45 years	9134	35.5
46-65 years	5397	21.0
>65 years	4045	15.7
Total	25740^a^	100.0

^a^Note: Patient age was not recorded for four calls

Of 23,254 patients, most patients only used the service once in 2014 (94.0%, *n* = 22,070 patients), 1,057 patients used the service twice (4.5%) and 360 patients (1.5%) called three or more times.

### Caller characteristics

In 2014, 73.1% (*n* = 18,808) of medication-related calls were made by patients themselves and 14.1% (*n* = 3,837) were made by a parent of the patient (Table 2). The majority of calls (77.2%, *n* = 19,881) were made by female callers. Aboriginal and Torres Strait Islander people made 947 calls (3.7% of all medication-related calls).

**Table 2:** Callers relationship to patients

Callers relationship to patients	Number of calls	Percentage (%)
Self	18,808	73.1
Parent	3,837	14.9
Other relatives	1,204	4.7
Employee	1,166	4.5
Carer	588	2.3
Other	141	0.6
Total	25,744	100.0

There were 23,254 people who made medication-related calls in total. Most people made a single medication-related call (93.2%, *n* = 21,683 callers), 5.0% (*n* = 1,165) called twice, and 1.8% (*n* = 406) made three or more (up to 19 times) medication-related calls.

### Call profile

The number of medication-related calls was distributed evenly across weekdays. Although there was little variation in call numbers between the days of the week, Wednesdays (15.0% of calls, *n* = 3,861 calls) were the busiest and Sundays (12.8%, *n* = 3,285 calls) were the quietest.

**Fig. 3:**
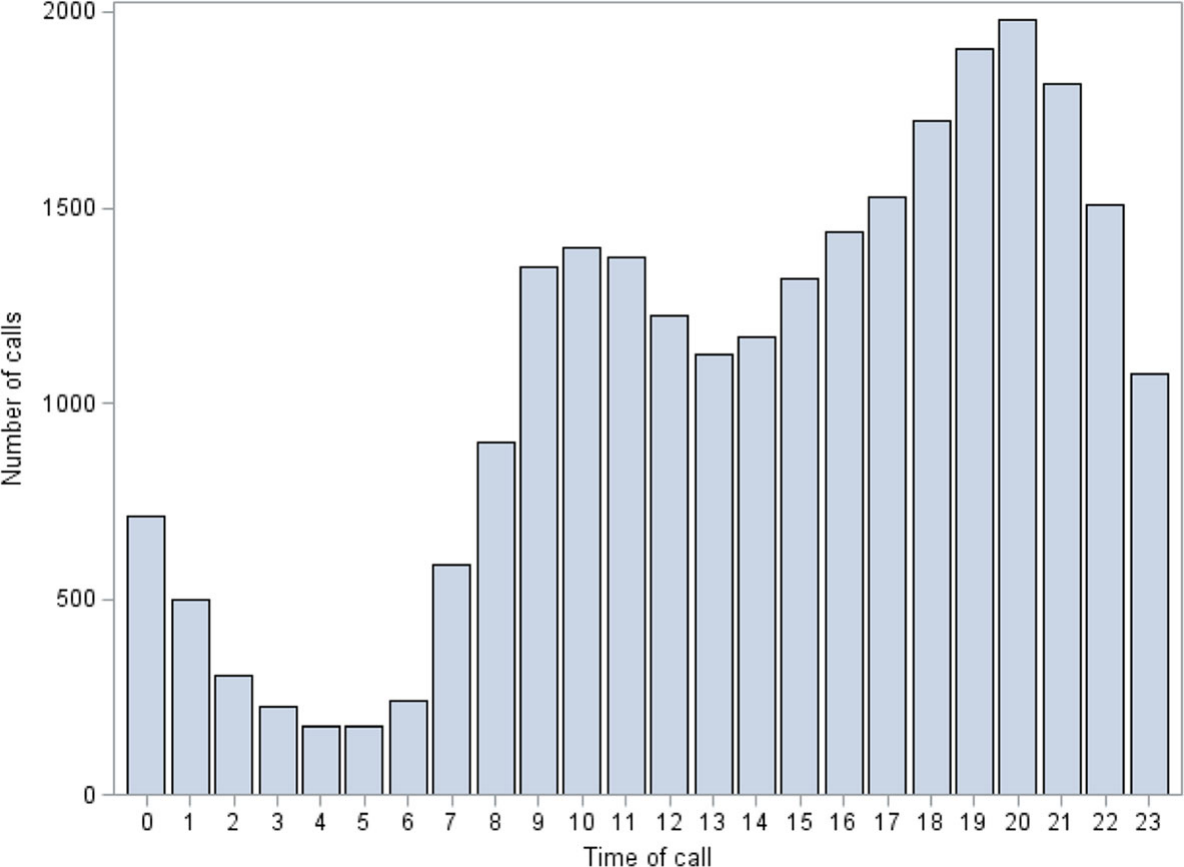
Distribution of 25, 744 medication-related calls by hour of the day

The volume of calls fluctuated over the day (Fig. 3). More than half of the calls were made after business hours, i.e. from 17:00 to 8:00 (56.1%, *n* = 14,449 calls). The busiest hours were 17:00–22:00 with 34.8% (*n* = 8,952 calls) of all medication-related calls made during this period.

The duration of calls varied from 2 to 55 min with an average length of 10 min (median 9 with the interquartile range (IQR) from 7 to 12 min). There were few differences in the duration of calls by other related characteristics, including patient demographics (such as age, gender, and state), caller relationship to patient, final care advice given by the nurses, caller’s age, gender or time of call.

### Callers’ original intentions and care advice given by the nurses

**Fig. 4:**
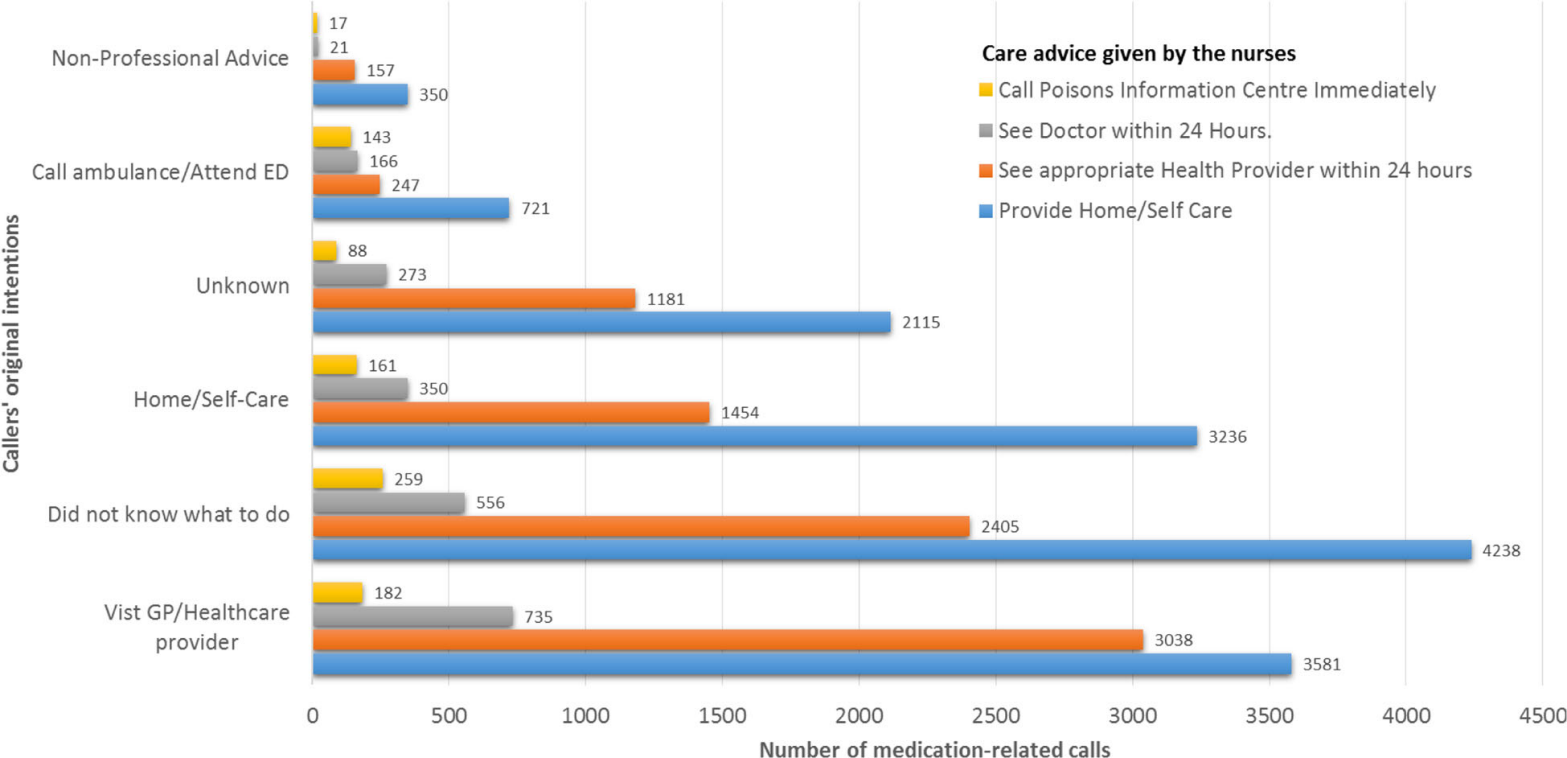
Medication-related calls (*n* = 25,744) by caller original intentions and care advice given by the nurses

Callers received care advice from the healthdirect helpline nurses. More than half of the calls (55.4%, *n* = 14,263; Fig. 4) ended with the advice to home/self-care and 41.3% of calls (*n* = 10,629) ended with the advice to see a health provider or doctor within 24 h. Advice to see another health provider includes advice to speak to a local pharmacist. While no caller was advised to call an ambulance or attend an ED, 850 callers (3.3%) were transferred to the Poisons Information Centre immediately.

For nearly one third of calls (29.4%, *n* = 7,458), callers reported they did not know what to do prior to calling the service. More than half of these calls (56.8%, *n* = 4,238) ended with the advice of home/self-care and 39.7% of these calls (*n* = 2,961) ended with the advice to see a health provider or doctor within 24 h. Importantly, 259 of these calls (3.5%) ended with a transfer to the Poisons Information Centre immediately.

For another nearly one third of calls (29.4%, *n* = 7,537), callers had planned to visit the GP or another healthcare provider prior to calling the healthdirect helpline. Nearly half of these callers (47.5%, *n* = 4,238) were advised to stay home or administer self-care.

For one fifth of calls (20.3%, *n* = 5,202), callers had planned to stay home or administer self-care. Nearly two thirds of these calls (62.2%, *n* = 3,236) ended with the same recommendation, however, 3.1% (*n* = 161) of these callers were transferred to the Poisons Information Centre immediately.

There were 1277 calls (5.0%) where the caller had originally intended to attend an ED or call an ambulance but other advice was given for these calls.

### Medications involved

Of 55,653 healthdirect helpline calls in November 2014, 1,835 (3.4%) were classified as medication-related by nurses. Of these calls, 204 (11.2%) had no medication information included in the presenting problem data and 380 medication names (15.0% of all medications identified) were unable to be extracted and classified despite efforts by the research team. The remaining 1,631 calls (88.8%) had up to 12 medications mentioned in a call. More than half of calls (55.3%) involved only one medication.

**Fig. 5:**
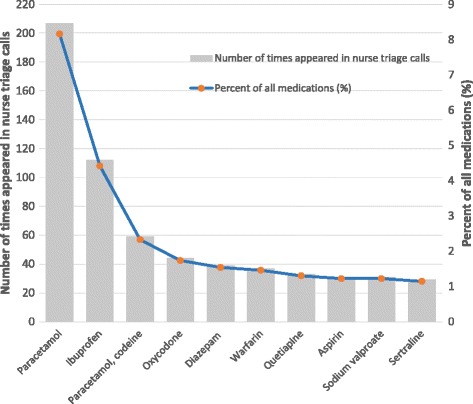
Ten most frequent generic medication names

We identified 2536 medications from our analysis of the free text data for calls made during November. We were able to classify 2,156 (85.0%) medications into 384 generic names. The most frequent medications enquired about were paracetamol (*n* = 207 times; 8.2% of all medications involved in medication-related calls; Fig. 5), followed by ibuprofen (*n* = 112, for 4.4% of medications), and paracetamol and codeine (*n* = 59, for 2.3% of medications).

**Fig. 6:**
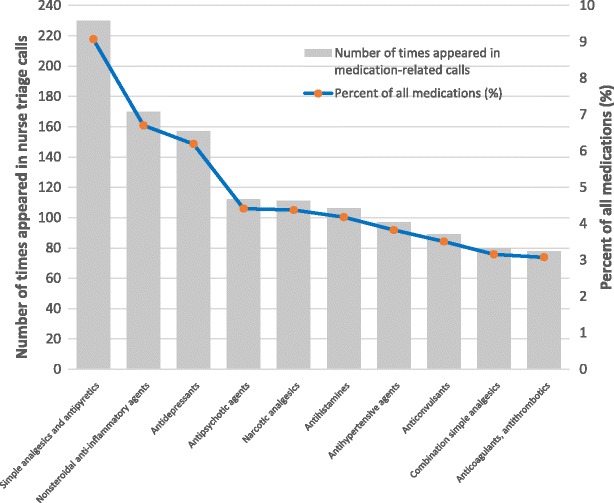
Ten most frequent medication therapeutic classes in the medication-related calls

We were able to classify 2,299 (90.7%) medications into 118 therapeutic classes. Table 3 shows the 10 most frequent therapeutic classes and provides examples of medications. The most frequent classes were simple analgesics and antipyretics (*n* = 230 times; 9.1% of all medications involved in medication-related calls; Fig. 6), followed by non-steroidal anti-inflammatory agents (*n* = 170, for 6.7% of medications), and antidepressants (*n* = 157, for 6.2% of medications).

**Table 3:** Ten most frequently appearing therapeutic classes and examples of medications

Therapeutic class	Examples of medications
Simple analgesics and antipyretics	Paracetamol
Combination simple analgesics	Paracetamol & codeine; paracetamol, codeine & doxylamine; ibuprofen & codeine
Narcotic analgesics	Fentanyl; oxycodone; morphine; codeine phosphate
Nonsteroidal anti-inflammatory agents	Ibuprofen; celecoxib; diclofenac; naproxen; piroxicam
Antipsychotic agents	Aripiprazole; risperidone; olanzapine; zuclopenthixol; haloperidol
Antidepressants	Citalopram; venlafaxine; amitriptyline; doxepin; dothiepin
Anticonvulsants	Gabapentin; lamotrigine; phenytoin; carbamazepine; sodium valproate
Antihypertensive agents	Ramipril; irbesartan; irbesartan & hydrochlorothiazide; nifedipine; diltiazem
Anticoagulants, antithrombotics	Warfarin; clopidogrel; aspirin (≤100 mg); apixaban
Antihistamines	Cetirizine; promethazine; dexchlorpheniramine; fexofenadine

## Discussion

### A quick and easily accessible telenursing triage and advice helpline

Medication-related calls constituted 3.8% of total calls to the healthdirect helpline service and made up the largest individual category of calls. More than half of these callers (55.4%) were advised by nurses to stay home and administer self-care. Of 7,459 calls where the callers did not know what to do prior to calling the helpline, 56.8% were advised to stay home and administer self-care. A significant proportion of calls ended with the advice of home/self-care providing reassurance to callers and patients that more serious intervention was not required. The average length of a call was 10 min indicating that on average the nurse telephone helpline was able to provide quick advice to patients who had medication queries.

The results provide some evidence that advice received diverted unnecessary use of more costly health services. Of 1,277 calls (5%) where callers had originally intended to ring an ambulance or to attend an ED, none were advised to do so. Our findings are consistent with a recent study on medication-related queries received by an ‘after hours GP helpline’, which showed that less than 2.3% of callers were directed to the ED despite 10.6% of people originally calling with this intention [24]. For one third of calls (29.4%, *n* = 7,537), callers had planned to visit the GP or another healthcare provider and nearly half of these callers were advised to stay home and administer self-care. Importantly, there was also evidence that the service was able to quickly direct a small proportion (3.5%) of callers requiring urgent, specialised advice, who rang uncertain of what to do, to the Poison Information Centre immediately. However, a limitation of this study was that we have no information on patients’ actual compliance with the advice.

### Planning and managing the national telenursing service

The results from this study could be useful for the service provider to plan and manage services according to the community need for the medication advice. The need for medication advice appeared to be consistent across day of the week while it fluctuated over hour of the day. About one third of calls (34.8%) were made between 17:00 and 22:00 when there is less access to a caller’s regular GP or local pharmacist.

Reported disadvantages of telephone consultation include both over- and under-utilisation of the service by different population groups [25], nuisance calls [3], and poor compliance with advice [21]. In this study, the majority of patients (67.9%) and callers (77.2%) were female, consistent with several other studies of queries to pharmacists [10, 11, 13, 15, 16].

Aboriginal and Torres Strait Islander people comprise 3% of the Australian population [26]. They appear to be utilising the healthdirect helpline service well, making 3.7% of calls, even though a previous study found that many Aboriginal people were reluctant to take medication and unlikely to seek out medication advice [27].

The medications about which callers most frequently enquired correspond with health conditions of high prevalence in the population. A 2001 study [28] found that 20% of females and 17% of males in New South Wales, the most populous state in Australia, experienced chronic pain. The most common medications about which advice was sought were simple analgesics and antipyretics, which are generally available without a prescription and used to treat pain and fever, as well as cold and flu symptoms. The second most commonly enquired about class, non-steroidal anti-inflammatory agents, is used to manage pain, inflammation and fever. Narcotic analgesics were the fifth most frequently enquired about medication type.

Antidepressants and antipsychotics were the third and fourth most frequently enquired about medications respectively in this study. A National Review in 2014 [29] found that approximately 20% of Australian adults can experience mental health problems each year, potentially explaining the high rate of enquiries about these medications. The 2007 National Survey of Mental Health and Wellbeing [30] found that 45.5% of Australians will experience a mental health problem at some point during their lives, and one in seven people will experience clinical depression during their lives. Pohjanoksa-Mäntylä et al. reported that people with mental illness have expressed dissatisfaction with the information received from pharmacists and physicians, relating to their medication treatments, and this may reflect that antidepressant users have unmet information needs [15]. However, we could not identify that inadequate information was one of the reasons for the calls since there was limited information recorded in the healthdirect helpline database.

### Medication information needs in community

There was a disparity between the most frequently enquired about medications in our study and the most commonly dispensed drugs to Australians, as reported by the Pharmaceutical Benefits Scheme (PBS) [31]. The most common medication, and the third most common were both hypolipidaemic agents, which did not appear in our top ten classes. Paracetamol was ranked the sixth most commonly used drug in the Australian community for 2014 by the PBS in their findings while paracetamol was the most enquired about medicine in our study. However, PBS medications only reflect medications provided on a prescription from a medical practitioner which are subsidised by the Australian Government and thus excludes medications purchased over the counter, as well as those that are not subsidised by the Government. Our analysis of the medications that are most enquired about could offer valuable insights into medication information needs in the community. It is of interest that the most frequent class of medications enquired about, simple analgesics and antipyretics, is one which is largely available over the counter without a prescription. It is possible that the easy availability and widespread usage of such medications, without intervention by GPs or pharmacists, may contribute to a lack of information in the community about them.

Prescription medications in Australia come with detailed consumer information leaflets, including information about dosage, interactions and side effects, yet research suggests these leaflets are not the preferred source of information for consumers. In a German study, Stahl et al. interviewed pharmacy customers regarding their use of product information leaflets [32]. They observed that the product information leaflet was only read selectively, if at all, and that consumers preferred to receive medication information from their GP. It was also found that if consumers experienced a potential adverse drug effect, their first action would be to contact a doctor or pharmacist, rather than to read the product information. While there may be many reasons why people might prefer to speak with a healthcare professional than consult a written information leaflet, it is likely that some calls would be averted if written information was better targeted and consumers utilised the information already available to them. It may also suggest that some callers may be seeking more than just information, but also reassurance.

## Limitations

While call characteristics were examined for the entire year 2014, only the November call data were examined in relation to the specific medication advice given, which may somewhat limit the representativeness of those findings due to seasonal variation in medications used. This may particularly be the case regarding medications such as antihistamines, however, seasonal variation is unlikely to significantly affect the rate of use of most other medications.

## Conclusion

To our knowledge, this is the first study to provide a profile of medication-related calls to a telenursing triage and advice service and the medications involved. The telenursing triage and advice helpline offered quick and easily accessible advice to patients with medication-related queries. Medication advice provided by telenursing services can effectively refer more serious cases to the Poisons Information Centre. The service provided reassurance to patients/callers, especially those who did not know what to do, and also potentially diverted patients from attending an ED or general practitioner where it was not medically necessary.

## Appendix

### MIMS therapeutic medication classes

**Table:**

Alimentary System • Hyperacidity, reflux and ulcers • Antispasmodics and motility agents • Laxatives • Antidiarrhoeals • Digestive supplements and cholelitholytics • Topical anorectal medication Cardiovascular system • Antihypertensive agents • Beta-adrenergic blocking agents • Diuretics • Antiarrhythmic agents • Antiangina agents • Hypolipidaemic agents • Cardiac inotropic agents • Adrenergic stimulants, vasopressor agents • Peripheral vasodilators • Antimigraine preparations • Anticoagulants, antithrombotics • Haemostatic agents • Fibrinolytic agents • Other cardiovascular agents Central Nervous System • Sedatives, hypnotics • Antianxiety agents • Antipsychotic agents • Antidepressants • Other central nervous system agents • Movement disorders • Anticonvulsants • Antiemetics, antinauseants Analgesia • Narcotic analgesics • Simple analgesics and antipyretics • Combination simple analgesics Musculoskeletal System • Nonsteroidal anti-inflammatory agents • Antirheumatoid agents • Muscle relaxants • Rubefacients, topical analgesics/NSAIDs • Agents used in gout and hyperuricaemia • Neuromuscular agents Endocrine and Metabolic Disorders • Adrenal steroid hormones • Gonadal hormones • Pituitary hormones • Insulin preparations • Hypoglycaemic agents • Thyroid hormones and antithyroid agents • Agents affecting calcium and bone metabolism • Anabolic agents • Haemopoietic agents • Other endocrine and metabolic agents Allergic Disorders • Antihistamines • Antiallergy preparations Ear, Nose and Oropharynx • Topical otic medication • Topical nasopharyngeal medication • Topical oropharyngeal medication Eye • Topical ocular anti-infective preparations • Topical ocular steroid preparations • Glaucoma preparations • Mydriatics, cycloplegics, miotics • Ocular decongestants, anaesthetics, anti-inflammatories • Ocular lubricants • Other ophthalmic medication Skin • Emollients, antipruritics and protective preparations • Psoriasis, seborrhoea and ichthyosis • Acne, keratolytics and cleansers • Wart and corn removers • Topical corticosteroids • Topical antibiotics • Topical antifungals • Topical antiparasitics • Topical antiseptics, anti-infectives • Leg ulcer preparations • Other dermatological preparations Surgical Preparations • Anaesthetics - local and general • Neuromuscular blocking agents • Cholinergic and anticholinergic agents • Plasma volume expanders • Surgical antiseptics and applications Contraceptive Agents • Combined oral contraceptive agents • Progestogen only contraceptive agents • Spermicidal contraceptives • Contraceptive devices Nutrition • Infant formulas • Supplemental and enteral nutrition • Parenteral vitamins, minerals and nutrition • Oral and parenteral electrolytes • Anorectics and weight reducing agents	Genitourinary System • Urinary antiseptics, alkalinisers and acidifiers • Bladder function disorders • Agents acting on the uterus • Topical vaginal medication • Erectile dysfunction agents • Other genitourinary agents Infections and Infestations • Penicillins • Cephalosporins • Tetracyclines • Macrolides • Quinolones • Aminoglycosides • Other antibiotics and anti-infectives • Antifungal agents • Antiviral agents • Antituberculotics and antileprotics • Antimalarials • Anthelmintics Neoplastic Disorders • Alkylating agents • Antimetabolites • Vinca alkaloids • Antibiotic cytotoxics • Hormonal antineoplastic agents • Other antineoplastic agents • Noncytotoxic and supportive therapy Immunology • Vaccines • Immunoglobulins • Immunomodifiers • Antisera, antivenoms Respiratory System • Expectorants, antitussives, mucolytics, decongestants • Bronchospasm relaxants • Bronchodilator aerosols and inhalations • Preventive aerosols and inhalations • Other respiratory agentsOther respiratory agents Poisoning, Toxicity and Drug Dependence • Detoxifying agents, antidotes • Agents used in drug dependence Diagnostic agents • Radiographic agents and bowel preparations • Agents for urinalysis • Ovulation, pregnancy detection agents • Other diagnostic agents Vitamins and Minerals • Vitamins (single agents) • Minerals • Fatty acid supplements • Fat soluble vitamins • B Group vitamins with other agents • Multivitamins and minerals • Tonics • Children’s vitamins • Iron Herbal and other complementary medicines • Herbal gastrointestinal preparations • Herbal circulatory system preparations • Herbal nervous system preparations • Herbal analgesics and anti-inflammatories • Women’s supplements • Men’s supplements • Herbal respiratory preparations • Herbal skin preparations • Herbal urinary tract preparations • General well-being, multiple use preparations, others

## Abbreviations

ED: Emergency department
GP: General practitioner
ICT: Information and communication technologies
IQR: Interquartile range
MIMS: Monthly index of medical specialties
PBS: Pharmaceutical benefits scheme
